# Letter from Dr. W. N. Cote

**Published:** 1865-01

**Authors:** 


### LETTER FROM DR. W. N. COTE.

SEPTEMBER 9TH, 1864.

ANIMALCULAR ORIGIN OF DISEASE.—In a paper recently addressed to the Academy of Sciences, Drs. Leplard and Jaillard examine whether microzoaria are the real causes of the diseases in which their presence has been ascertained in the human body. You are aware that in their endeavors to explain the origin and propagation of contagious disorders, physicians have often attributed these affections to the existence of invisible animalcules, parasites, or impalpable ferments. This hypothesis was extremely plausible, and received a superstructure of various medical theories, but these had still to be verified, for while it was necessary to prove that there exist cryptogamous plants and infusoria capable of engendering diseases. Various attempts have been made to solve this important problem, and observers of unquestionable merit, considering all kinds of virus to be ferments, and ferments to be animated beings, have not hesitated to attribute to infusoria the development of carbuncle, typhus fever, and other affections, because they had found such microscopic animalcules in the blood of subjects that had died of these maladies. Our authors are of opinion that such assertions are, at least, premature, and ought to have been supported by more direct evidence than that as yet adduced. Thus, instead of inoculating animals, as some have done, with the blood of subjects laboring under carbuncles, a blood in which the microscope can detect but very few of the elements it contains, they think it more advisable to operate with bacteria free from all accessories, which might either rightly or wrongly be suspected of being the active principles of the disease. Bacteria, as your readers are aware, are infusoria of the genus vibrio; under the microscope they have the appearance of threads, and they are developed in all liquids containing animal matter in a state of decomposition. It is, therefore, very easy to procure these animalcules, and our authors have performed a series of experiments, by injecting liquids containing them into the cellular tissues, and even the veins of rabbits, dogs, and other animals. These experiments show that the introduction of bacteria or vibrios into the blood causes no virulent disorders, although the putrified substances introduced may produce the effects of poison.

International Medical Congress.—I have now before me the draft of the convention proposed by a committee of the International Medical Congress at Geneva, for general adoption, and agreed to, and to which I referred in my preceding letter. I think it well to place it before your readers.

"The undersigned plenipotentiaries assembled in congress at Geneva, have adopted the following arrangements to be observed in case of hostilities breaking out between their respective countries:—

1. Ambulances and military hospitals shall be recognized as neutral, and as such, protected and respected by the belligerents as long as they shall contain sick or wounded.

2. All the sanitary staff, including physicians and surgeons, apothecaries, attendants, officials, and generally all persons attached to the service of hospitals and ambulances, shall be considered neutralized.

3. The above-mentioned persons shall be permitted, even after occupation by the enemy, to continue to fulfil their duties in the hospital or ambulance to which they are attached, as long as shall be necessary, after which they shall be allowed to depart without being in any way hindered or inconvenienced.

4. These persons, however, shall not be permitted to remove any articles but those which are their own private property. All materials employed in the arrangement of the ambulance or hospital will remain subject to the rights of war.

5. The inhabitants of the country, who may be employed in the transport of the wounded, or in bringing them assistance upon the field of battle, shall be equally respected, and remain entirely free.

6. Soldiers badly wounded, whether already received into the ambulances and hospitals, or whether picked up upon the field, shall not only have there hurts attended to irrespective of their nationality, but shall also not be made prisoners. They may return to their homes, upon condition of not again taking up arms during the course of the campaign.

7. A safe conduct and, if necessary, the costs of the route, shall be handed to soldiers mentioned in the preceding article, when after cure, they leave the place where they have been nursed.

8. Articles requisite for the sick and the persons attached to the ambulance, shall be supplied to the army in occupation, which shall be subsequently repaid the outlay shown to have been incurred, by receipts furnished for the purpose.

9. A distinctive and uniform armlet shall be adopted by the sanitary officials and staff of all armies. An identical flag shall also be employed in all countries, to distinguish ambulances and military hospitals. The armlet and flag shall be those agreed upon by the International Conference which met at Geneva in 1863, (a red cross upon a white ground.)

10. Those persons, who, without being entitled to wear the armlet, shall adopt it to enable them to act as spies, shall be punished with the full rigor of military law.

11. Similar stipulations to the preceding, relative to naval warfare, shall form the object of a further convention between interested powers.

Oxygen and Ozone.—M. St. Edme publishes certain experiments, from which it appears that oxygen separated by the electrolytic process from a binary compound is not ozonized, and that this state only manifests itself when the decomposing power of electricity has been brought to bear on a double chemical affinity. M. St. Edme is consequently inclined to suppose that ozone is but a different dynamic state of oxygen, and not a chemical or physical transformation.—*Med. & Surg. Reporter.*

